# Nusinersen Treatment for Spinal Muscular Atrophy: Retrospective Multicenter Study of Pediatric and Adult Patients in Kuwait

**DOI:** 10.3390/neurolint16030047

**Published:** 2024-06-04

**Authors:** Asma AlTawari, Mohammad Zakaria, Walaa A. Kamel, Nayera Shaalan, Gamal Ahmed Ismail Elghazawi, Mohamed Esmat Anwar Ali, Dalia Salota, Amr Attia, Ehab Elsayed Ali Elanany, Osama Shalaby, Fatema Alqallaf, Vesna Mitic, Laila Bastaki

**Affiliations:** 1 Pediatric Department, Neurology Unit, Al Sabah Hospital, Shuwaikh Industrial 70050, Kuwait; 2 Pediatrics Department, Al Adan Hospital, Hadiya 47000, Kuwait; 3 Neurology Department, Ibn Sina Hospital, Shuwaikh Industrial 70050, Kuwait; 4 Neurology Department, Faculty of Medicine, Beni-Suef University, Beni-Suef 62521, Egypt; 5 Pediatric Department, Al Jahra Hospital, Al Jahra 003200, Kuwait; 6 Pediatric Department, Neurology Unit, Mubarak Hospital, Jabriya 46300, Kuwait; 7 Pediatric Department, Al Farwaniya Hospital, Al Farwaniya 85000, Kuwait; 8 Kuwait Medical Genetics Center, Shuwaikh Industrial 70050, Kuwait

**Keywords:** spinal muscular atrophy, neuromuscular disease, nusinersen, antisense oligonucleotide, pediatrics

## Abstract

Spinal muscular atrophy is a neuromuscular genetic condition associated with progressive muscle weakness and atrophy. Nusinersen is an antisense oligonucleotide therapy approved for the treatment of 5q spinal muscular atrophy in pediatric and adult patients. The objective of this clinical case series is to describe the efficacy and safety of nusinersen in treating spinal muscular atrophy in 20 pediatric and 18 adult patients across six treatment centers in Kuwait. Functional motor assessments (Children’s Hospital of Philadelphia Infant Test of Neuromuscular Disorders, Hammersmith Functional Motor Scale Expanded, and Revised Upper Limb Module) were used to assess changes in motor function following nusinersen treatment. The safety assessment involved clinical monitoring of adverse events. The results demonstrate clinically meaningful or considerable improvement in motor performance for nearly all patients, lasting over 4 years in some cases. A total of 70% of patients in the pediatric cohort and 72% of patients in the adult cohort achieved a clinically meaningful improvement in motor function following nusinersen treatment. Additionally, nusinersen was well-tolerated in both cohorts. These findings add to the growing body of evidence relating to the clinical efficacy and safety of nusinersen.

## 1. Introduction

Spinal muscular atrophy (SMA) is a progressive neuromuscular disorder and the most common genetic cause of infant mortality [1]. The global incidence of SMA is 1 in 10,000 live births [2]. However, the incidence of SMA in the Middle East is reportedly 1–20 in 10,000 live births [3,4,5,6]. It is thought that higher rates of consanguinity in the region may account for the increased incidence and carrier frequency [2,7]. A recent study in Saudi Arabia reported an SMA carrier frequency of 2.6%, which is higher than the reported global frequency of 1.25–2% [8].

SMA is an autosomal recessive disease characterized by muscle atrophy caused by insufficient production of survival motor neuron (SMN) protein [9]. This results in degradation of motor neurons in the anterior horn of the spinal cord [10]. SMA is of five types: 0 to IV in decreasing order of severity and increasing order of typical age of onset [11]. Around 60% of patients with SMA have the severe, infantile-onset type I form, associated with the inability to sit independently, loss of motor skills, progressive weakness, failure to thrive, scoliosis, dysphagia, and breathing problems [12]. SMA type II is associated with onset between 6 and 18 months, developmental delays, and loss of motor skills, with the ability to sit independently when placed [10]. Both SMA types I and II are associated with substantial impairment and mortality in the context of natural history of disease [13,14]. In adults, SMA type III (associated with onset between 18 months and 18 years and independent ambulation) or IV (associated with onset after 18 years and normal ambulation) can limit mobility and cause weakness and fatigue [15]. However, there is thought to be a degree of heterogeneity in terms of clinical presentations and disease manifestations [16].

Insufficient SMN protein production arises from homozygous deletion or a pathogenic mutation of the *SMN1* gene located on chromosome 5q13 [17]. This results in complete reliance on the similar *SMN2* gene. However, only around 10% of *SMN2* transcripts generate functional SMN protein [18]. It is thought that disease severity correlates with the number of copies of *SMN2*, with fewer gene copies associated with more severe phenotypes [19,20]. The variability in *SMN2* gene copy number among individuals contributes to the spectrum of clinical severity observed in SMA patients.

Recent advances in genetic research and therapeutic approaches have significantly impacted the management of SMA. The introduction of disease-modifying therapies such as nusinersen has shifted the treatment paradigm, offering new hope for improved outcomes in SMA. These novel therapies aim to address underlying genetic defects, thereby altering the disease course and enhancing the quality of life of patients and their caregivers. Despite these advancements, challenges remain in ensuring equitable access to these treatments, particularly in regions with limited healthcare resources. This underscores the need for continued research and data collection to optimize treatment strategies.

Nusinersen is an antisense oligonucleotide approved for the treatment 5q SMA in pediatric and adult patients [21]. It increases production of SMN protein in the central nervous system by altering the splicing of *SMN2* to promote exon 7 inclusion, thus improving motor neuron survival [22,23]. Studies have demonstrated that nusinersen improves motor function in SMA and increases survival rates in type I patients [7,22,24]. Nusinersen is also effective in treating symptoms of SMA in adults, and real-world data are increasingly providing an understanding of its potential benefits and tolerability in adult SMA [21,25].

The aim of this case series is to describe the clinical efficacy and safety of nusinersen in 38 patients with SMA (20 pediatric and 18 adults) across six treatment centers in Kuwait. This study seeks to contribute to the growing body of real-world evidence on the use of nusinersen, particularly in regions with higher genetic predispositions to SMA, and to highlight the practical challenges and outcomes associated with its administration in diverse clinical settings.

## 2. Materials and Methods

### 2.1. Study Design and Participants

In this multicenter case series, we describe 20 pediatric and 18 adult patients with SMA who received nusinersen at Al Sabah, Al Adan, Al Jahra, Al Farwaniya, Mubarak, and Ibn Sina Hospitals in Kuwait (treatment initiation period between July 2016 and February 2022). We discuss the clinical efficacy and safety of nusinersen within these patient groups. All participants had a diagnosis of 5q SMA confirmed via genetic documentation. SMA-type classification was not used to exclude patients. The age ranges of patients were 2 to 12 years in the pediatric cohort and 18 to 55 years in the adult cohort, and 14 of the 38 included patients were female.

### 2.2. Nusinersen Treatment

All patients received nusinersen via intrathecal injection as per the current standard therapy recommendations [26]. In the loading phase, four 12 mg doses were administered (first three doses in 14-day intervals and the fourth dose 30 days after the third). Subsequently, 12 mg maintenance doses were administered every 4 months. For the purpose of this case series study, the day of administration of the first loading dose was considered the first day of treatment initiation for each patient.

### 2.3. Functional and Safety Assessments

Among the pediatric cohort of 20 patients, motor function was evaluated in 14 patients via the Children’s Hospital of Philadelphia Infant Test of Neuromuscular Disorders (CHOP-INTEND) at baseline and several points after initiation of nusinersen treatment. The CHOP-INTEND is a validated 16-item scale, with a maximum score of 4 for each checklist component [27]. For the remaining six patients in the pediatric cohort (all with SMA types II or III), gross motor function was evaluated via the Hammersmith Functional Motor Scale Expanded (HFMSE) tool, which consists of 33 items scored 0, 1, or 2, giving a maximum score of 66 [28]. Within the adult cohort, the Revised Upper Limb Module (RULM) assessment was used as the vast majority of patients were non-ambulant at the time of presentation. This is also a validated tool used in SMA types II and III, with 19 scorable items mostly graded using a 3-point system and a maximum score of 37 for each upper limb [29]. Safety assessment involved the clinical monitoring of adverse events and complications.

### 2.4. Data Analysis

Linear trendlines (moving average models) were used to illustrate changes in functional motor outcomes for each patient. Changes in motor performance for pediatric patients 1–7 and patients 8–14 are visualized on separate graphs for clarity. The same applies for adult patients 21–29 and 30–38. Due to the retrospective nature of this study, there are some inconsistencies in the data reported, such as missing baseline functional assessment scores for some patients. A clinically meaningful improvement in functional motor performance was defined as an increase of ≥4 points, ≥3 points, and ≥2 points in the CHOP-INTEND, HFMSE, and RULM score, respectively [23,24,30,31,32,33,34]. Considerable improvement in functional motor performance was defined as any increase in the CHOP-INTEND, HFMSE, or RULM score ˂4 points, ˂3 points, and ˂2 points, respectively. Clinical stabilization was defined as maintenance of the CHOP-INTEND, HFMSE, or RULM score, and motor decline was defined as any decrease in the CHOP-INTEND, HFMSE, or RULM score.

## 3. Results

### 3.1. Patient Characteristics

A total of 20 pediatric and 18 adult patients with SMA were included in this case series study. Among the pediatric cohort, 45% of the patients were female. 40% were diagnosed with type I SMA, 40% with type II SMA, and 20% with type III SMA. 10% had one copy of *SMN2*, 50% had two copies, and 10% had three copies. 50% had a confirmed family history of SMA.

Among the adult cohort, 28% of the patients were female. 11% were diagnosed with type II SMA and 89% were diagnosed with type III SMA. 17% had two copies of *SMN2*, 44% had three copies, and 11% had four copies. 72% had a confirmed family history of SMA and 94% of cases were associated with parental consanguinity. Patient characteristics (Tables S1–S3) are summarized in Table 1 and Table 2.

### 3.2. Functional Motor Studies

**Figure 1:**
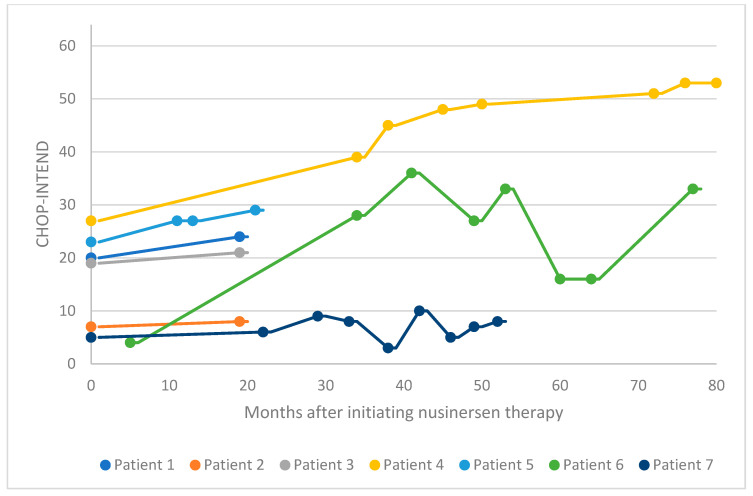
CHOP-INTEND scores for patients 1–7 after nusinersen treatment initiation. CHOP-INTEND, Children’s Hospital of Philadelphia Infant Test of Neuromuscular Disorders.

**Figure 2:**
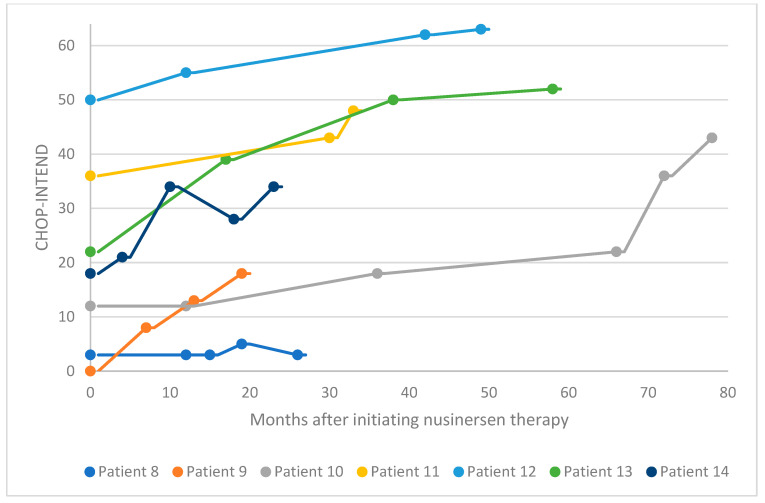
CHOP-INTEND scores for patients 8–14 after nusinersen treatment initiation. CHOP-INTEND, Children’s Hospital of Philadelphia Infant Test of Neuromuscular Disorders.

In the pediatric cohort, motor function was evaluated in 14 patients via CHOP-INTEND assessment at baseline and several points following treatment initiation (Figure 1 and Figure 2). In 13 patients, CHOP-INTEND assessments demonstrated a clinically meaningful or considerable improvement in functional motor performance after treatment initiation compared with baseline, and one patient was stabilized. Note that fluctuations were observed in the motor performance of patients 6, 7, and 14 over time. This may be attributed to these patients missing doses or receiving delayed doses due to national travel restrictions imposed in Kuwait following the COVID-19 pandemic. Within this subgroup of patients, the average increase in CHOP-INTEND from the earliest recorded assessment point to the latest was 13.7. A proportion of 71% of these patients experienced a clinically meaningful improvement in motor performance (≥4 CHOP-INTEND points) following nusinersen treatment, and no patients experienced motor decline associated with SMA progression.

**Figure 3:**
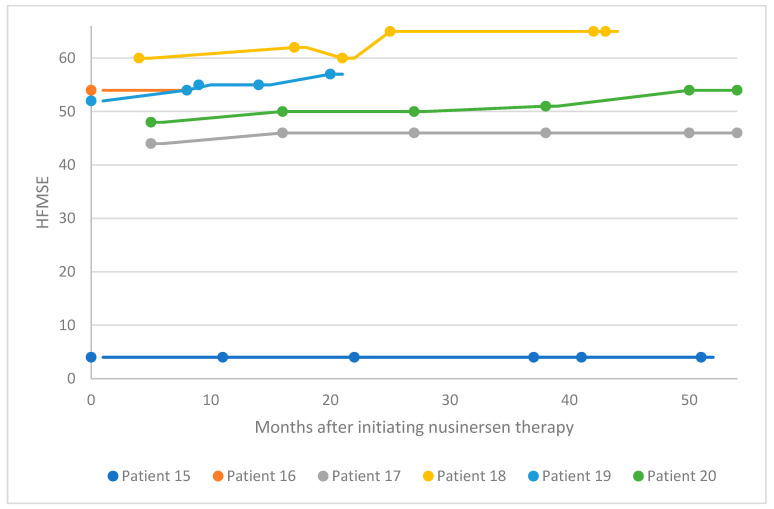
HFMSE scores for patients 15–20 after nusinersen treatment initiation. HFMSE, Hammersmith Functional Motor Scale Expanded.

For six patients in the pediatric cohort, motor function was evaluated via HFMSE assessment (Figure 3). Four patients in this subgroup experienced a clinically meaningful improvement in functional motor performance, whereas one patient experienced a considerable improvement. Patient 15 had a low baseline HFMSE score and exhibited stabilization over 50 months of treatment. Within this subgroup, the average increase in HFMSE from the earliest to the latest assessment point was 3.5. A proportion of 67% of patients in this subgroup experienced a clinically meaningful improvement in motor performance (≥3 HFMSE points) following nusinersen treatment, and no patients experienced motor decline associated with SMA progression. Overall, in the pediatric cohort (i.e., patients 1–20), 70% of patients experienced a clinically meaningful improvement in motor function following nusinersen treatment, and 20% of patients experienced a considerable improvement in motor function.

**Figure 4:**
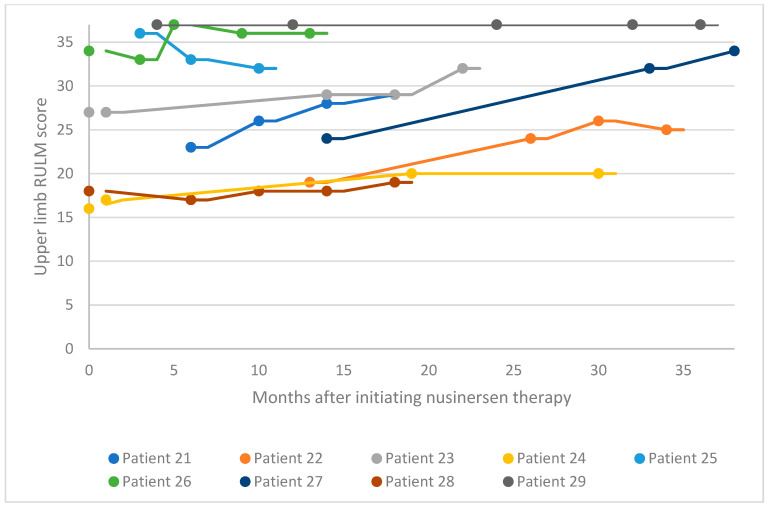
Upper limb RULM scores for patients 21–29 after nusinersen treatment initiation. RULM, Revised Upper Limb Module.

**Figure 5:**
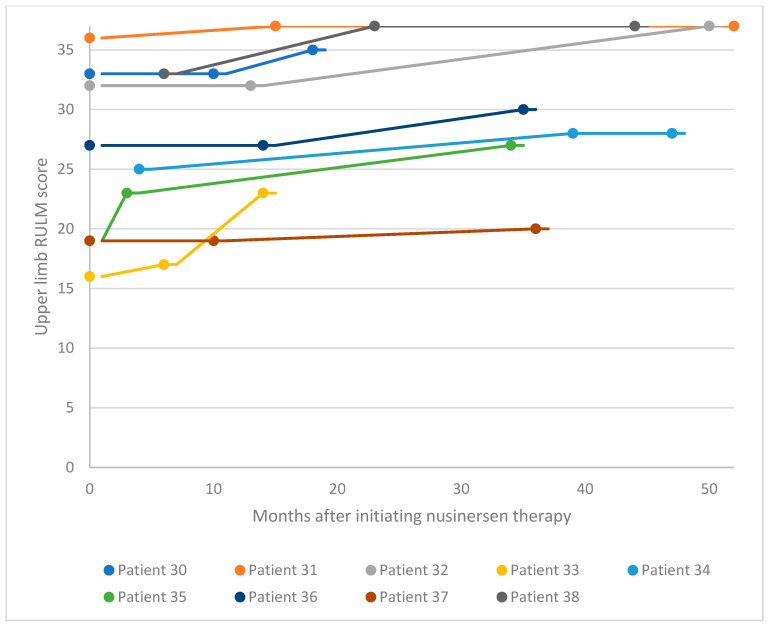
Upper limb RULM scores for patients 30–38 after nusinersen treatment initiation. RULM, Revised Upper Limb Module.

For the adult SMA cohort, RULM assessment was used to evaluate changes in motor performance following treatment initiation (Figure 4 and Figure 5). Of the 18 patients in the adult cohort, 13 experienced clinically meaningful improvement in functional motor performance, three experienced considerable improvement, and one was clinically stabilized. Just one patient with a high baseline RULM score exhibited a slight decline in motor performance associated with SMA disease progression. This is thought to be related to the patient missing or receiving delayed doses of nusinersen due to national travel restrictions implemented in Kuwait following the COVID-19 pandemic. Within the adult cohort, the average increase in RULM score was 7.3 from the earliest to the latest assessment point. A proportion of 72% of patients in the adult cohort experienced a clinically meaningful improvement in motor performance (≥2 RULM points) following nusinersen treatment, and 17% of patients experienced a considerable improvement.

### 3.3. Clinical Presentations and Motor Milestones

11 patients of the 20 in the pediatric cohort were diagnosed with SMA and initiated nusinersen treatment in infancy. In addition, 10 of these patients exhibited clinically meaningful or considerable motor function improvement, and six were capable of sitting independently. Nine patients in the pediatric cohort were diagnosed after the age of 12 months. Of them, three were entirely wheelchair-bound, two were limited to assisted walking, and four were capable of unassisted walking.

Among the adult SMA cohort of 18 patients, 14 were non-ambulant at the time of initial presentation. Patient 26, who was ambulant at the initial presentation, had HFMSE scores of 41, 49, and 52 at five, nine, and 13 months of nusinersen treatment, respectively. On the six-minute walk test, this patient scored 250 m at three months of treatment and 335 m at five months. All patients in the adult cohort responded well to nusinersen therapy, with no deterioration in motor function observed.

### 3.4. Safety Outcomes

From the pediatric cohort, three patients were admitted for chest infections on more than one occasion. One patient developed severe scoliosis and underwent corrective surgery. Six other patients also developed mild or moderate scoliosis. After 2 years of nusinersen treatment, one patient experienced mild elevations in prothrombin time and activated partial thromboplastin time (17.9 and 37.0 s, respectively). This resolved naturally within 3 months. In relation to drug administration, one patient occasionally experienced post-injection headaches and another experienced fibrous tissue formation at the site of lumbar puncture, which was detected during a physical examination. Overall, there were no serious adverse events related to nusinersen use in the pediatric cohort. 

Among the adult SMA patient cohort, nusinersen was very well-tolerated. The only adverse safety event was the development of mild restrictive lung disease in one patient.

## 4. Discussion

In this retrospective, multicenter study in Kuwait, we investigated the effects of nusinersen in 38 patients with SMA. There were 20 patients in the pediatric cohort, eight diagnosed with SMA type I, eight diagnosed with SMA type II, and four diagnosed with SMA type III. In the adult cohort of 18 patients, two were diagnosed with SMA type II and 16 were diagnosed with SMA type III. This is the first retrospective clinical case series of nusinersen for SMA in Kuwait, and its findings add to the growing body of real-world efficacy and safety data pertaining to antisense oligonucleotide therapy.

The retrospective design, while not ideal due to some data inconsistencies (missing baseline motor scores and treatment interruptions), provides valuable insights into the real-world application of nusinersen. The data reflect practical challenges faced in routine clinical settings, including logistical issues, patient referral delays, inconsistent monitoring practices across treatment centers, and resource constraints exacerbated by the COVID-19 pandemic. Unlike controlled clinical trials, this study captures the variability in management and adherence, offering an accurate representation of the efficacy and safety of nusinersen in a real-world context. Retrospective case series are crucial in understanding how nusinersen performs outside of highly controlled clinical trial environments, thus providing a comprehensive view of its performance and limitations in routine clinical practice.

In the pediatric cohort in this study, 70% of patients achieved clinically meaningful improvement in motor function following nusinersen treatment, as assessed by CHOP-INTEND of HFMSE. Two exceptions were patients with low baseline functional motor scores, who were clinically stabilized and showed no further motor deterioration. Three other patients experienced fluctuations in motor performance over time, which is thought to relate to missed doses and interruptions in physical therapy due to national travel restrictions imposed following the COVID-19 pandemic. In the adult cohort, 72% of patients achieved clinically meaningful improvement in motor function following nusinersen treatment, as assessed by RULM. Two exceptions were patients with very high baseline RULM scores, in whom the ceiling effect was observed.

In terms of safety, nusinersen was well-tolerated within both cohorts in this study, which is consistent with recently published observational studies [21,35,36]. The occurrence of chest infections and mild restrictive lung disease is likely a feature of the disease rather than an adverse effect of nusinersen treatment. Chest infections are a common complication in SMA patients due to weakened respiratory muscles and impaired cough reflexes, which increase susceptibility to respiratory infections [37]. Similarly, the development of scoliosis, which is a well-documented feature of SMA attributed to muscle weakness and imbalances, is also thought to be a consequence of disease activity as opposed to a side effect of nusinersen [38,39]. In addition, transient coagulation abnormalities have also been reported in other studies of nusinersen, though they are considered rare and mild [40,41,42]. Furthermore, post-lumbar puncture headaches are a known complication of intrathecal drug administration, including nusinersen [43,44]. Although fibrous tissue formation is not a typical outcome of lumbar punctures, it is thought to have occurred in one patient as a mild and transient inflammatory response to the procedure. Overall, there were no drug-related serious adverse events in this study, which aligns with safety findings reported in large-scale clinical trials of nusinersen.

This case series demonstrates improved motor function in patients with SMA following nusinersen treatment in real-world clinical settings in Kuwait. The findings are congruent with results from the ENDEAR phase III clinical trial, which investigated the efficacy and safety of nusinersen in SMA in 121 infants [23]. In the ENDEAR clinical trial, several infants who received nusinersen experienced a clinically meaningful improvement in neuromuscular function, assessed by CHOP-INTEND. A proportion of 73% of infants in the nusinersen group had an increase of at least one point in their CHOP-INTEND score compared to 3% in the control group. Infants in the nusinersen group also had significantly higher likelihoods of event-free and overall survival compared to the control group, despite also having greater burden-of-disease characteristics at baseline [23]. Interestingly, the proportion of infants who experienced any increase in CHOP-INTEND score in the ENDEAR trial (73%) was similar to the proportion of infants who achieved a clinically meaningful improvement in the CHOP-INTEND score (i.e., ≥4 points) (71%) in our study. This is partly because patients in the ENDEAR trial were followed up until the end-of-trial visit on day 394, whereas patients in our clinical case series study were followed up over much longer durations on average.

In the CHERISH phase III clinical trial, investigators studied the efficacy and safety of nusinersen in 126 children with SMA who had symptom onset after six months of age. At 15 months of treatment, 57% of children experienced a clinically meaningful improvement in motor function (i.e., ≥3 point improvement in HFMSE) [24]. In our study, 67% of children had an HMFSE score improvement of ≥3 points following nusinersen treatment, though this analysis pertains to a small group of just six patients. In the SHINE open-label extension study, nusinersen demonstrated improvement or stabilization in motor function measures for up to nearly 6 years [45]. Similarly, the findings presented in this case series demonstrate sustained motor function improvement or stabilization over long-term periods (i.e., over four years for eight patients included).

The improved functional motor performance of patients treated with nusinersen is thought to be associated with enhanced function and/or survival of motor neurons caused by increased SMN protein production. It has previously been demonstrated that the pharmacology of nusinersen is consistent with its intended mechanism of action, with increases in the amount of functional *SMN2* messenger RNA transcript and SMN protein having been observed [12]. Therefore, the drug reaches its target tissues and promotes exon 7 inclusion during gene splicing in the central nervous system. It is also thought that the number of *SMN2* gene copies affects the SMA phenotype, with fewer copies associated with more severe disease [20,46]. Indeed, similar associations between the SMA type and *SMN2* copy number were noted within the cohorts of patients included in this study.

There are a few limitations associated with this case series. Firstly, baseline functional motor data were unavailable for some patients who initiated nusinersen treatment outside of Kuwait. For these patients, we report the earliest recorded functional motor score obtained a few months after therapy was initiated. Had baseline functional scores been available for all patients, even greater average improvements in motor performance may have been observed. Additionally, clinical measures solely relied on assessments of motor function, and as such, the benefits of nusinersen in non-motor domains were not captured. While quality of life was monitored informally during clinical visits through discussions with patients and their families, structured quality of life assessment tools were not used consistently across treatment centers due to logistical and resource constraints. Furthermore, we only report HFMSE and six-minute walk test data for one out of four adult ambulant patients. For the other adult ambulant patients, the HFMSE tool and six-minute walk test were used inconsistently (e.g., partial assessment of HFMSE checklist items due to specific challenges with these patients). These data do not meet our standards for quality and consistency and were therefore excluded from the study. Despite these limitations, this study is meaningful as it is the first to report on the real-world clinical efficacy and safety of nusinersen in patients with SMA in Kuwait.

Overall, this case series describes promising clinical outcomes following nusinersen therapy in SMA. This is particularly relevant to Kuwait, a country with higher-than-average levels of consanguinity and disease incidence. Further clinical data generation and research is recommended in the country to identify novel protocols for optimal disease management and improved patient outcomes.

## 5. Conclusions

This case series demonstrates that nusinersen is generally well-tolerated in pediatric and adult patients with SMA in Kuwait. Most patients experienced a clinically meaningful improvement in functional motor performance following treatment initiation, with benefits sustained for over 4 years in eight patients. Further large-scale studies are warranted to confirm the findings reported.

## Figures and Tables

**Table 1:** Patient characteristics of the pediatric SMA cohort.

Number of Patients	Proportion of Female Patients (%)	SMA Type (%)	Number of *SMN2* Gene Copies (%)	Proportion with a Family History of SMA (%)
20	45	Type I: 40	1: 10	50
		Type II: 40	2: 50	
		Type III: 20	3: 10	
		Type IV: 0	Unknown: 30	

SMA, spinal muscular atrophy.

**Table 2:** Patient characteristics of the adult SMA cohort.

Number of Patients	Proportion of Female Patients (%)	SMA Type (%)	Number of *SMN2* Gene Copies (%)	Proportion with a Family History of SMA (%)	Proportion with Parental Consanguinity (%)
18	28	Type I: 0	2: 17	72	94
		Type II: 11	3: 44		
		Type III: 89	4: 11		
		Type IV: 0	Unknown: 28		

SMA, spinal muscular atrophy.

## Data Availability

The data supporting the findings of this study are available within the article.

## Supplementary Materials

The following supporting information can be downloaded at: https://www.mdpi.com/article/10.3390/neurolint16030047/s1, Table S1: Individual patient characteristics and outcomes for pediatric patients 1–14. Table S2: Individual patient characteristics and outcomes for pediatric patients 15–20. Table S3: Individual patient characteristics and outcomes for adult patients 21–38.
