# Correction: AlTawari et al. Nusinersen Treatment for Spinal Muscular Atrophy: Retrospective Multicenter Study of Pediatric and Adult Patients in Kuwait. *Neurol. Int*. 2024, *16*, 631–642

**DOI:** 10.3390/neurolint17010004

**Published:** 2025-01-02

**Authors:** Asma AlTawari, Mohammad Zakaria, Walaa A. Kamel, Nayera Shaalan, Gamal Ahmed Ismail Elghazawi, Mohamed Esmat Anwar Ali, Dalia Salota, Amr Attia, Ehab Elsayed Ali Elanany, Osama Shalaby, Fatema Alqallaf, Vesna Mitic, Laila Bastaki

**Affiliations:** 1 Pediatric Department, Neurology Unit, Al Sabah Hospital, Shuwaikh Industrial 70050, Kuwait; 2 Pediatrics Department, Al Adan Hospital, Hadiya 47000, Kuwait; 3 Neurology Department, Ibn Sina Hospital, Shuwaikh Industrial 70050, Kuwait; walaaneuro@yahoo.com (W.A.K.); nayerashaalan@gmail.com (N.S.); 4 Neurology Department, Faculty of Medicine, Beni-Suef University, Beni-Suef 62521, Egypt; 5 Pediatric Department, Al Jahra Hospital, Al Jahra 003200, Kuwait; 6 Pediatric Department, Neurology Unit, Mubarak Hospital, Jabriya 46300, Kuwait; 7 Pediatric Department, Al Farwaniya Hospital, Al Farwaniya 85000, Kuwait; vesnaoct@gmail.com; 8 Kuwait Medical Genetics Center, Shuwaikh Industrial 70050, Kuwait

## Additional Affiliation

In the published publication [1], there was an error regarding the affiliation for the following authors:

- Walaa A. Kamel. In addition to affiliation 3—Neurology Department, Ibn Sina Hospital, Shuwaikh Industrial 70050, Kuwait, the updated affiliation should include 4—Neurology Department, Faculty of Medicine, Beni-Suef University, Beni-Suef 62521, Egypt.
- The name of the third author (Walaa Kamel) was not mentioned correctly in the manuscript.

The correct name is as follows: Walaa A. Kamel.

- The name of the ninth author (Ehab Elsayed Ali Elanay) was not mentioned correctly in the manuscript.

The correct name is as follows: Ehab Elsayed Ali Elanany.

The authors state that the scientific conclusions are unaffected. This correction was approved by the Academic Editor. The original publication has also been updated.
