# Supplementary material for: Nusinersen Treatment for Spinal Muscular Atrophy: Retrospective Multicenter Study of Pediatric and Adult Patients in Kuwait
Source: Neurol Int. 2024 Jun 4;16(3):0. doi: 10.3390/neurolint16030047 (PMC11206794; doi:10.3390/neurolint16030047)
Supplement: Supplementary file 1 [file neurolint-16-00047-s001.zip › neurolint-2991539-supplementary.pdf]

Supplementary Information

Table S1. Individual patient characteristics and outcomes for pediatric patients 1-14.

**Case number**	**Gender**	**SMA type**	**Number of *SMN2* gene copies**	**Family history of SMA**	**CHOP-INTEND (baseline)**		**CHOP-INTEND (most recent follow-up)**		**Treatment response**	**Adverse events**
					**Months after treatment initiation**	**Score**	**Months after treatment initiation**	**Score**		
1	Female	I	1	(+)	0	20	19	24	Clinically meaningful	Chest infections, scoliosis, elevations of PT and aPPT
2	Male	II	1	(+)	0	7	19	8	Considerable	Chest infections, scoliosis
3	Female	II	2	(+)	0	19	19	21	Considerable	Chest infections, scoliosis
4	Female	I	2	(+)	0	27	80	53	Clinically meaningful	None
5	Female	I	2	(+)	0	23	21	29	Clinically meaningful	None
6	Male	I	2	(-)	5	4	77	33	Clinically meaningful	None
7	Male	I	2	(-)	0	5	52	8	Considerable	None
8	Female	I	2	(-)	0	3	26	3	Stabilization	None
9	Female	I	Unknown	(-)	0	0	19	18	Clinically meaningful	None
10	Male	II	Unknown	(-)	0	12	78	43	Clinically meaningful	None
11	Male	II	2	(-)	0	36	33	48	Clinically meaningful	None
12	Female	II	Unknown	(-)	0	50	49	63	Clinically meaningful	None
13	Male	II	Unknown	(-)	0	22	58	52	Clinically meaningful	None
14	Male	I	2	(-)	0	18	23	34	Clinically meaningful	Scoliosis

aPPT, activated partial thromboplastin time. CHOP-INTEND, Children's Hospital of Philadelphia Infant Test of Neuromuscular Disorders. PT, prothrombin time. SMA, spinal muscular atrophy.

Table S2. Individual patient characteristics and outcomes for pediatric patients 15-20.

Case number	Gender	SMA type	Number of *SMN2* gene copies	Family history of SMA	HFMSE (baseline)		HFMSE (most recent follow-up)		Treatment response	Adverse events
					Months after treatment initiation	Score	Months after treatment initiation	Score		
15	Male	II	2	(-)	0	4	51	4	Stabilization	Scoliosis
16	Female	III	Unknown	(+)	0	54	20	57	Clinically meaningful	Scoliosis
17	Male	II	2	(+)	5	44	54	46	Considerable	Scoliosis
18	Male	III	3	(+)	4	60	43	65	Clinically meaningful	Scoliosis
19	Female	III	Unknown	(+)	0	52	20	57	Clinically meaningful	Scoliosis, headache
20	Male	III	3	(+)	5	48	54	54	Clinically meaningful	Scoliosis, lumbar region fibrous tissue formation

HFMSE, Hammersmith Functional Motor Scale – Expanded. SMA, spinal muscular atrophy.

Table S3. Individual patient characteristics and outcomes for adult patients 21-38.

Case number	Gender	SMA type	Number of *SMN2* gene copies	Family history of SMA	Parental consanguinity	RULM (baseline)		RULM (most recent follow-up)		Treatment response	Adverse events
						Months after treatment initiation	Score	Months after treatment initiation	Score		
21	Male	III	3	(+)	(+)	6	23	22	32	Clinically meaningful	None
22	Male	III	Unknown	(+)	(+)	13	19	34	25	Clinically meaningful	None
23	Male	III	4	(+)	(+)	0	27	22	32	Clinically meaningful	None
24	Male	III	2	(+)	(+)	0	16	30	20	Clinically meaningful	Restrictive lung disease
25	Female	III	3	(+)	(+)	3	36	10	32	Motor decline	None
26	Female	III	4	(-)	(+)	0	34	13	36	Clinically meaningful	None
27	Male	III	Unknown	(-)	(+)	14	24	38	34	Clinically meaningful	None
28	Female	III	Unknown	(+)	(+)	0	18	18	19	Considerable	None
29	Male	III	2	Unknown	Unknown	4	37	36	37	Stabilization	None
30	Male	III	Unknown	(+)	(+)	0	33	18	35	Clinically meaningful	None
31	Male	III	3	(-)	(+)	0	36	52	37	Considerable	None
32	Male	III	3	(+)	(+)	0	32	50	37	Clinically meaningful	None
33	Male	III	Unknown	(+)	(+)	0	16	14	23	Clinically meaningful	None
34	Male	III	2	(+)	(+)	4	25	47	28	Clinically meaningful	None
35	Male	III	3	(+)	(+)	0	19	34	27	Clinically meaningful	None
36	Male	II	3	(+)	(+)	0	27	35	30	Clinically meaningful	None
37	Female	III	3	(+)	(+)	0	19	37	20	Considerable	None
38	Female	II	3	(-)	(+)	6	33	44	37	Clinically meaningful	None

RULM, Revised Upper Limb Module. SMA, spinal muscular atrophy.
